# Agricultural Holdings and Slaughterhouses’ Impact on Patterns of Pathological Findings Observed during Post-Mortem Meat Inspection

**DOI:** 10.3390/ani11051442

**Published:** 2021-05-18

**Authors:** Johannes Klinger, Beate Conrady, Marina Mikula, Annemarie Käsbohrer

**Affiliations:** 1 Unit for Veterinary Public Health and Epidemiology, University of Veterinary Medicine, Veterinärplatz 1, 1210 Vienna, Austria; Johannes.klinger@vetmeduni.ac.at (J.K.); bcon@sund.ku.dk (B.C.); 2 Department of Veterinary and Animal Sciences, Faculty of Health and Medical Sciences, University of Copenhagen, 1870 Frederiksberg C, Denmark; 3 Federal Ministry of Social Affairs, Health, Care and Consumer Protection, Radetzkystraße 2, 1030 Vienna, Austria; m.mikula@a1.net

**Keywords:** slaughter, post-mortem meat inspection, pathological findings, permutation ANOVA, pig production

## Abstract

**Simple Summary:**

The pig sector represents one of the most economically important farming sectors in the European Union. Farmers and their veterinarians are responsible for ensuring animal health as well as sending healthy animals for slaughter. At slaughter, official veterinarians conduct meat inspections to ensure that only meat fit for human consumption enters the food processing chain. The data collected during meat inspections can provide valuable information about herd health for the farmers, the attending veterinarians and veterinary authorities. The aim of this study was to describe the data and analyse whether these data are suitable to be used as a feedback system for farmers to enable them to improve their herd management. Data used comprised all meat inspection findings in 2016 from 4.6 million pigs originating from 9172 agricultural holdings (farms) and slaughtered in 66 slaughterhouses in Austria. Analysis showed that diseases of the lung (pneumonia, 21.9%) and of the liver (milk spots, 19.9%) were most frequently detected. The agricultural holdings, the slaughterhouses and the time periods (quarter) had an influence on the observed occurrence and composition of the prevalence of post-mortem findings. Furthermore, within the slaughterhouses, the recorded patterns of pathological findings differed, which points towards the need for further harmonisation to provide high-quality feedback to farmers and veterinarians.

**Abstract:**

Meat inspection data can provide valuable information about herd health to producers, veterinarians and veterinary authorities and can be used as a feedback system for farmers to improve their herd management. The aim of this study was to analyse the influence of agricultural holdings, slaughterhouses and time periods (quarters) on the occurrence and composition of the prevalence of post-mortem findings of 4 million pigs slaughtered in Austria in 2016, by applying a permutation multivariate analysis of variance. Pneumonia (21.9%) and milk spots (19.9%) were the most frequently recorded conditions. Our analysis indicated a statistically significant influence of all three considered factors (agricultural holdings, slaughterhouses and periods) on the prevalence of post-mortem findings. The observed prevalence could not only be explained by the differences between the farms of origin and slaughterhouses but also by the variability within the slaughterhouses. Much of the explained variance of the prevalence was due to differences between producers (mean R^2^ = 0.61), followed by slaughterhouses (mean R^2^ = 0.19) and period (mean R^2^ = 0.05). To meet the demand for a valid feedback system to farmers and attending veterinarians, a robust and ideally more detailed recording of frequent pathologies, especially those affecting the respiratory tract and the liver, should be developed.

## 1. Introduction

The pig sector represents one of the most economically important farming sectors in the European Union, making up 8.5% (total value: EUR 34.4 billion) of the European Gross Value Added (GVA) for agriculture. In total, 257 million pigs were slaughtered in the European Union in 2016, resulting in 23.4 million metric tons of pig meat and an annual average consumption of 45.9 kg per EU inhabitant. According to Regulation (EU) 2017/625, carcases and offal of all slaughtered pigs are subject to post-mortem inspection in the slaughterhouses to decide whether the meat is fit for human consumption. Further details for the performance of official controls can be found in Commission implementing regulation (EU) 2019/627. The frequency of pathological findings is a valuable indicator for the assessment of health and safety issues, which provides information about herd health [1] and can be used to support farmers wanting to improve their herd management [2]. Yet a substantial lack of suitable data on the frequency of many diseases and conditions affecting food animals exists in Europe [3]. Furthermore, records on meat inspection findings of pig carcases are considered valuable indicators of financial losses due to morbidity of the animals [4,5,6,7]. Ferraz et al. [8] showed that a high percentage of lung lesions at slaughter lead to economic losses of up to USD 6.55 (EUR 5.40) per pig slaughtered. Other studies indicated that the frequency of pathological findings depends not only on the farm management and related health status of the pigs [9,10,11], but also on the veterinarians who carried out the meat inspections [12,13,14,15,16,17], slaughterhouses [12], and seasonal occurrence of certain lesions [9,12,18,19]. However, most other studies assessed the influencing factors in individual slaughterhouses [17,20] or for single pathological findings [18].

The most significant slaughterhouse findings are pathologies associated with the respiratory system, such as pneumonia, pleuritis, foreign body in the lung, and pericarditis. Additionally, cranio-ventral pulmonary consolidation, which is strongly linked to enzootic pneumonia (*Mycoplasma hyopneumoniae*), caudo-dorsal lung alterations, connected to an infection with *Actinobacillus pleuropneumoniae*, and pleuritis have been reported to be the most frequent pathological findings in Northern Italy [21]. Reports from Germany have shown that interstitial pneumonia (93%) is the most common lung-related pathology and pleuritis is the least common (5%). Pathologies regarding the liver (e.g., milk spots, hepatitis) are also of great significance and are the second most frequent organs affected after respiratory tract infections [22]. Similar observations were also made in Italy [19] and in the UK [23]. *Trichinella spiralis*, which has led to fatal infections in humans in Austria in the past [24], was not reported in slaughterhouses in Austria in recent years [25], although its presence is routinely examined in all pig carcases.

To the authors’ knowledge, the analysis presented here is the first to consider the prevalence of all post-mortem pathological findings recorded in 66 slaughterhouses for an entire national pig population over a one-year period. The aims of this study were (i) to provide information about the prevalence of the individual pathologies and abnormalities recorded at post-mortem meat inspection, (ii) to identify potential influencing factors (i.e., agricultural holdings, slaughterhouses and quarters) on the prevalence of these findings, and to determine the variation of the prevalence explained by these factors.

## 2. Materials and Methods

### 2.1. Data

**Figure 1:**
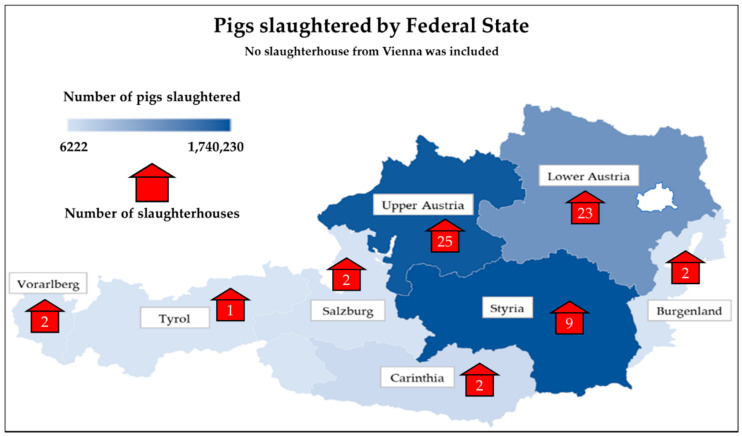
Number of slaughterhouses and slaughtered pigs by Austrian federal state based upon the dataset analysed.

We used data on all pathological findings from pigs slaughtered in 2016 in Austrian slaughterhouses, which were digitally recorded during routine post-mortem meat inspections, regardless of whether the entire carcase or individual organs were condemned. In total, our dataset included 4,604,716 pigs, covering all types (i.e., with respect to age and sex) of pigs and production systems. These data consisted of 3.6 million records of post-mortem findings in pigs supplied in 146,853 consignments from 9172 agricultural holdings to 66 Austrian slaughterhouses in 2016. The number of slaughterhouses and slaughtered pigs per federal state are shown in Figure 1. More than half of all producers (60.9%) in this dataset routinely provided consignments of pigs to only one slaughterhouse, while the remainder used two or more different slaughterhouses (Supplementary Figure S1). The mean annual number of consignments delivered for slaughter per producer was four and consisted of an average of 110 animals. The mean annual number of pigs slaughtered per producer and per slaughterhouse were 502 (range: 1 to 42,035) and 69,768 (range: 519 to 452,390), respectively.

These data were provided by the Ministry of Labour, Social Affairs, Health and Consumer Protection in Austria and included the number and type of pathological findings per consignment and quarter, the number of pigs undergoing meat inspection per consignment, the unique registration number of each agricultural holding of origin (usually farm of origin) and slaughterhouse, the quarter of recording, and the location of the slaughterhouses (i.e., federal state). A consignment was defined as a group of pigs delivered from an individual pig producer to the same slaughterhouse per quarter. It may have consisted of one or more deliveries. All lesions found during meat inspection are recorded using a coding system, which has been laid down in national legislation (Meat Inspection Regulation). Recording several findings per animal was possible. Codes for recording post-mortem findings in Austria distinguished between condemnation of the entire carcase (D-codes) and trimming (E-codes). Welfare-related codes (C-codes) were excluded from this analysis, as they are considered ante-mortem findings. Note, A-, B- and C-codes document ante-mortem complaints.

To calculate the prevalence of each finding, the total number of slaughtered pigs per consignment was compared with the number of pigs with pathological findings recorded in this consignment. In total, six consignments were excluded from further analysis as the number of animals with findings exceeded the number of slaughtered animals in these consignments, which was not plausible. We grouped the pathological findings into five main categories taking into account a previously published categorisation system [26], according to topographic appearance as follows: Category 1—pathologies of the respiratory system and the heart, Category 2—pathologies of the abdominal organs, Category 3—pathologies of the skin and the locomotor system, Category 4—other pathologies and Category 5—slaughter-technique-induced abnormalities. For the assessment of influencing factors, the prevalence of pathological findings was statistically analysed for each of these five main categories individually by federal state.

### 2.2. Statistical Analysis

The (normality) distribution of the prevalence was checked for multivariate data in semi-parametric factorial designs with the corresponding Q-Q plots and residual vs. fitted plots and the Shapiro–Wilk Test. Due to the non-normal and zero-inflated (i.e., no pathological findings on pigs) distribution, as well as observed overdispersions by implementing generalised linear models for individual pathological findings, the data were analysed by applying a PERMANOVA (non-parametric permutational multivariate analysis of variance). PERMANOVA allows for a geometric partitioning of multivariate variation by using dissimilarly (distance) measures. We calculated the Bray–Curtis dissimilarity of the prevalence with the R function vegdist, and this distance matrix was applied as a response variable. In the first step, a PERMANOVA-model was calculated analysing the impact of the factors slaughterhouses, agricultural holdings and season on the proportion of animals with pathological findings. Similar to ANOVA, PERMANOVA also measures the sum of squares between and within each group and compares the between and within group variance using the F-test. The result for significance is, however, obtained by comparing the results from both the random permutations and F-test results. The distribution of the multivariate F-Statistic (hypothesis: no effect of the considered factors) is unknown [27]. Consequently, the Fisher’s F ratio and corresponding *p*-value were calculated using permutation techniques and the results are considered statistically significant at *p*-value < 0.05. In the second step, the model was rerun for each category separately. A pairwise comparison between the groups of a factor (i.e., slaughterhouse, animal holding, quarter) within each pathology category was then performed with the pairwiseAdonis function. The total sum of the distance is similar to the total variance and can be differentiated into variability between groups and the variability within groups [27]. Additionally, the variance (R^2^) explained by these factors on the prevalence data was analysed for each category and federal state. The homogeneity of the group variances (H-value) was analysed with the function betadisper for group disperations, a multivariate analogue of Levene′s test, followed by a permutation-based test of multivariate homogeneity of group dispersions. The H-value < 0.05 was interpreted as an indication of heteroscedasticity within a group of the considered factor.

Additionally, the Spearman rank correlation rho (ρ) was calculated between the prevalence of the individual pathological findings instead of the categories. The statistical analysis was implemented using the R statistical computing environment (Version 3.4.1 R Foundation for Statistical Computing, Vienna, Austria) using the package vegan, pairwiseAdonis, Hmisc, PerformanceAnalytics and heatmap [28,29].

## 3. Results

### 3.1. Prevalence of the Individual Pathologies and Abnormalities at Post-Mortem Meat Inspection

Table 1 shows the prevalence of the main post-mortem findings (Category 1 and 2) summarised for all slaughtered pigs. Overall, the findings of the D-categories which reflect condemnation of the carcase were reported at low frequencies, making up to 3.5% of all animals slaughtered. Details on findings for Categories 3–5, which were all reported at a prevalence of <1%, can be found in the Supplementary Table S1.

The highest prevalence of findings was identified for respiratory-system-related disorders (Category 1: 48.3%; n = 2.23 million), followed by abdominal-system-related disorders (Category 2: 23.8%; n = 1.10 million). For Categories 3 to 5, a prevalence of between 1.3% and 2.7% was recorded. Findings in the respiratory tract, the heart and the abdominal organs (Category 1 and 2) comprised 92.0% of all reports. The ten most frequent individual post-mortem reports comprised 93.2% of all recorded findings and were made up of six pathological findings in Category 1, milk spots and hepatitis in Category 2 and singular arthritis and singular abscess in Category 3. The most prevalent pathological findings were pneumonia (21.9%; n = 1.01 million), milk spots (19.9%; n = 0.92 million) and foreign body in the lung (14.5%; n = 0.67 million).

The geographical distribution of the prevalence of the lung and heart (Category 1) is shown in Table 2. For pneumonia and foreign body in the lung, recorded prevalence varied widely between federal states.

A strong correlation was identified between the prevalence of foreign body (Code D140, Category 4) and abnormalities due to certain slaughter techniques (Code D190, Category 5) (ρ = 0.75; *p*-value < 0.001) but not to any other code including foreign body in the lung (Code E191). Furthermore, moderate correlation between some E-codes, e.g., the prevalence of pneumonia (Code E167), pericarditis (Code E168), pleuritis (Code E169), hepatitis (Code E166) and milk spots (Code E085), was observed but not among any other D-codes (Supplementary Figure S2).

### 3.2. Potential Influencing Factors and Variance Explained

The overall model showed that the majority of variance in the prevalence of pathological findings can be explained by agricultural holdings (mean R^2^ = 0.61 range 0.23–0.89), followed by slaughterhouses (mean R^2^ = 0.19; range 0.03–0.47) and quarter (mean R^2^ = 0.05; range 0–0.21). Table 3 shows the influence of agricultural holding, slaughterhouse and quarter on the occurrence and composition of pathological prevalence per category and federal state (according to the location of the slaughterhouse).

The impact of slaughterhouse could not be assessed for all regions, as the number of slaughterhouses was either too low or the pathological findings for certain categories were not recorded in any animal. Similarly, in some regional subsets, data were not available for all four quarters. In Category 1, the agricultural holding had the highest impact (range for R^2^ = 33.0–66.2%) in six federal states, whereas the slaughterhouse had a higher impact in two federal states which also had the highest number of slaughterhouses included in the analysis, namely, Lower Austria and Upper Austria. In these two federal states, variance explained by slaughterhouses was 47.4% and 35.0%, respectively. In Category 2, the farm of origin had the highest impact (range for R^2^ = 41.0–76.9%) in all but one federal state (namely, Carinthia). In that state, more variance was explained by the slaughterhouses (48.3%). For Tyrol, 47.4% of the variance was explained by the farm of origin, but a significant difference was only confirmed for the two quarters included, which explains 19% of the variance in this data subset. In Category 3, most variance was explained by agricultural holdings in all regions (range for R^2^ = 30.5–79.0%), but this was not significant (*p* > 0.05) in two federal states. In Category 4, agricultural holdings explained most of the variance (range for R^2^ = 42.7–81.2%) in seven regions, but not in Styria (38.1% by slaughterhouses). In Category 5, most variance was explained by the agricultural holdings (range for R^2^ = 31.0–89.5%), but this was not significant in four out of the eight federal states analysed.

### 3.3. Potential Heteroscedasticity within the Analysed Factors

The significant influence was linked to both the prevalence and the composition of the pathological findings of Category 1–4. In Category 5 (slaughter techniques), the majority of agricultural holdings had no significant influence on the records of findings. For Categories 1 to 4, significant effects could not be fully assigned to agricultural holdings as significant variability was also identified within the slaughterhouses (see H-values < 0.05, Table 3). This reveals that in those federal states where producers have the highest impact, the slaughterhouses also influence the occurrence and composition of the pathological findings. Nonetheless, homogeneity of the group variance was identified for seven cases: the significant effect of the slaughterhouses located in Vorarlberg (Category 1), Carinthia (Categories 2, 4, 5), and Burgenland (Categories 1, 4, 5) on the composition of pathological findings in the respective categories can only be explained by the difference between the slaughterhouses and not due to the variation within slaughterhouses themselves (Table 3).

**Figure 2:**
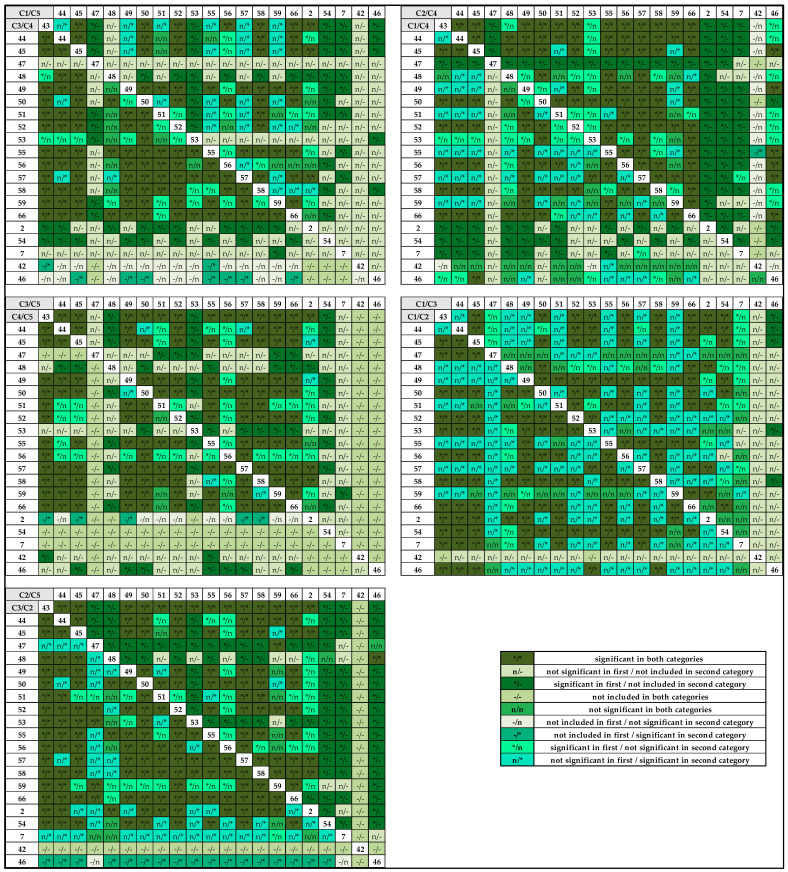
Pairwise comparison between slaughterhouses in Lower Austria stratified by the different pathological categories that were defined in Section 2.1. (Category 1–Category 5). C1 = Category 1, C2 = Category 2, C3 = Category 3, C4 = Category 4, C5 = Category 5; slaughterhouses are represented by random numbers (black numbers in white squares). * = significant (results were significantly different between the two slaughterhouses); n = not significant; - = not included (the comparison was not calculated). On each of the five Fs, the results of comparison of pathological findings between the individual two slaughterhouses for the two categories listed in the grey box are displayed (one result before, the other behind the slash). Above the diagonal line, the first two comparisons are displayed; below the diagonal line, the other two outcomes are displayed; for example: slaughterhouse 43 does not differ significantly from slaughterhouse 44 in Category 1 but does so in Category 5.

As an example, the pairwise comparison between the slaughterhouses in Lower Austria is shown in Figure 2. Among the 21 slaughterhouses in this region, up to 210 comparisons of category prevalence between slaughterhouses could be made. The number of significant pairs differed between the categories, with highest values for Category 2 (170/191 comparison: 89.0%). For Category 3, 69.6% (119 of 171) of slaughterhouse pairwise comparisons were statistically significant, while 65.6% of these significant slaughterhouse combinations were also statistically significantly in Category 4 (indicated as */* in Figure 2).

## 4. Discussion

### 4.1. Dataset

In total, 88.6% (n = 4,604,716) of all pigs slaughtered in Austria in 2016 were included in our dataset. The remaining slaughter pigs can be assigned to home-related meat production by smallholders [30]. Thus, a broad basis was available to describe the national prevalence of pathologies and abnormalities recorded during post-mortem meat inspection in Austrian slaughterhouses [3]. Understanding the validity of results from meat inspection as well as the possible influencing factors is important to draw conclusions beneficial for various stakeholders along the food chain. While it is important for pig producers and their attending veterinarians to obtain more information on herd health, it is also vital for slaughterhouse managers and policy makers to understand differences in slaughter processes and data recording on which to base further improvement [17,31].

### 4.2. Prevalence of the Individual Pathologies and Abnormalities

In contrast to other studies, where only a few selected findings or a small number of slaughterhouses were considered [19,20,31,32], our study considered data from 66 Austrian slaughterhouses using the electronic recording system (NB. small slaughterhouses without an electronic system and home-related slaughter (for own consumption) were not included) and all post-mortem meat inspection findings reflecting all pathologies and abnormalities which are required to be recorded in Austria.

Overall, findings of the D-categories, which reflect condemnation of the entire carcase, were reported at a frequency of 4.5%, with pleuritis (1.3%) being the most frequent individual finding. In contrast to our results, lower carcase condemnation rates have been reported in other studies, e.g., 0.03% in one Italian pig slaughterhouse [31], 0.24% in Portugal [33], or 0.35% in the United Kingdom [34]. However, the current study covered results from all slaughterhouses in the whole of Austria and, therefore, provides a fuller picture than previous studies.

As in our study, previously reported causes of carcase condemnation were relatively heterogeneous and included abscesses, peritonitis, septicaemia, cachexia, and pleuritis/pneumonia [31,35]. Much more frequently, alterations of specific organs were recorded by these authors. In line with our results, pathologies of the respiratory system (Category 1) and the liver (Category 2) have been reported elsewhere to be the most important and most common findings in routine meat inspections [9,10,21,22,23,36].

Post-mortem abnormalities of the respiratory tract were most common in our study (61.7% of all findings; 48.3% of all animals) as shown in several other studies [12,20,22]. Among these, findings such as pneumonia and pleuritis were most common [20,21], whereas, in our study, pneumonia (E167) was the most common finding with an overall prevalence of 21.9%. In a previous study by Schleicher et al. [17], which focused on one Austrian slaughterhouse, pleuritis was more common (34.7%) than pneumonia (30.4%); however, this may be due to separate counting of parietal and visceral alterations of the pleura (as several codes for pleuritis exist). In an Italian study, bronchopneumonic lesions suggestive of enzootic pneumonia were detected in 46.4% of the examined lungs. Again, figures for pleuritis were higher with 47.5% of lungs being classed as chronic pleuritic and 25.1% of the lungs showing dorsocaudal pleuritis suggesting that the animal had recovered from pleuropneumonia [21]. In contrast to this, in a German study, 77% of pig carcases exhibited pneumonia, whereas pleuritis was recorded in only 18% of these [37]. In a Danish study, chronic pleuritis was a common finding, with a prevalence of 19% among organic/free-range pigs and 24% among conventional pigs [38].

With respect to the finding “foreign body in the lung” (E191) allocated to the respiratory system (Category 1), other studies do not refer to similar findings, which may reflect the fact that such alterations are not recorded or have been excluded from further consideration. This may be caused by horizontal scalding machines still in use. Additionally, previous analysis of data from one Austrian slaughterhouse highlighted that the recording of blood aspiration and scalding water lungs is not sufficiently standardized in meat inspection recording [17]. Consequently, this may have contributed to the relatively high prevalence found here.

In our study, pericarditis and pluck adhesions were recorded at low frequencies only (2.3% and 1.3%, respectively). The figure for pericarditis is lower than in other studies, where rates between 3.2% and 10.8% have been reported [35,39]. In an Italian study, besides pericarditis, the second cause of heart condemnation was polyserositis, a code which does not exist in the Austrian system [31,40].

Findings associated with abdominal organs (30.4% of all findings) were frequent in the present study. These primarily concerned the liver, with milk spots (E085) as the second most common finding (19.9% of all animals). These liver lesions are usually, but not exclusively, a result of previous infection with the most important and most frequent porcine gastrointestinal macro-parasite, *Ascaris suum* [9,41]. In a British study involving two regional datasets, milk spots were present in 4.4% and 3.7% of the pigs [9]. In another study, where three pig health schemes were compared, the average annual prevalence of liver milk spots was highest in Northern Ireland (13–18%) compared with England (2–6%) and Scotland (3–8%) [18]. In a recent Italian study, milk spots were found in 30.86% of all pigs [31]. A study in Austria showed that 69.5% of organic pigs at slaughter were positive for *Ascaris suum* in their faeces, which reflects the fact that ascarid infection is still widespread in some porcine populations and production types [42]. A rise in ascarid prevalence within a farm should always lead to increased biosecurity measures and veterinary involvement. By contrast, the non-occurrence of such findings or a reduction over time may hint towards successful treatment or the absence of ascarids on a farm [40,43]. Codes for documenting lesions in the gastro-intestinal tract are currently not considered in the Austrian system. This information might be beneficial for describing the severity of an *Ascaris suum* infection within a herd, as *Ascaris suum* infection has a limited window of detection by means of milk spots. Thus, the overall prevalence of ascarid infection might be even higher than determined here [41].

Besides milk spots, hepatitis and perihepatitis (E166) were also recorded at a much lower frequency (3.3% of all animals) in Austrian slaughtered pigs. The reasons behind this record might be different. Perihepatitis can be due to infectious polyserositis, attributable to serious infections with *Actinobacillus pleuropneumoniae* or other primary polyserositis agents, or be secondary to lung infections [40]

In accordance with previous research from other countries, the vast majority of individual pathological findings occurred at a prevalence below 1% among all inspected carcases (38 of 47 findings (80.9%)). This is also true for pathologies related to the skin and the locomotor system (Category 3), other pathologies (Category 4) and slaughter-technique-induced abnormalities (Category 5), as none of the individual findings reached an overall prevalence of 1%. Among the lesions associated with the skin and the locomotor system, singular arthritis (0.6%) or polyarthritis (0.4%), as well as singular isolated abscess (<1%) and multiple abscesses (0.5%) were the most frequent findings, followed by skin parasites (0.3%). Lesions associated with the skin and the tail have previously been described as important welfare indicators in pigs, which are often recorded during meat inspection with a marked herd effect [20], but since C-codes have been excluded from this analysis and the Austrian meat inspection system is currently lacking an option to report tail lesions, no statement can be made on these lesions at this stage.

Several authors have highlighted that the methods of recording pathological findings varied between different EU countries [44]. Thus, the observed variations may be due to differences in judgement, the codes available for documentation or may also be attributed to the specific purposes of the research work if these overlap with the normal inspection activity [31]. In a comparative analysis involving three Italian slaughterhouses, classification of respiratory lesions differed considerably between meat inspectors, but when figures were combined comparable numbers of respiratory diseases were detected by all approaches [39].

### 4.3. Interactions between Findings

Recorded meat inspection findings might be linked to each other through underlying diseases or other common features. The highest correlation within consignments was observed in our study between two D-codes (foreign body, D140, Category 4, 0.4% and abnormalities due to certain slaughter techniques, D190, Category 5, 0.5%) which might reflect the fact that both codes are linked to issues with the slaughter process. In contrast to this, foreign body findings in the lung (E191, Category 1) were not associated with any other finding, including those reflecting slaughter technique difficulties. Analysis of regional patterns demonstrated that the frequency of this finding was extremely different among federal states (see Section 4.4 below).

In contrast to aspects relating to slaughter methods, medium correlation was observed between codes in Category 1 and Category 2, respectively, which can be understood as a reflection of animal health issues at farm level. Detailed analysis of our data showed correlations between records of pneumonia, pleuritis and pericarditis in the consignments. Pathologies potentially associated with septicaemia (e.g., pericarditis, peritonitis) appear interrelated, suggesting ongoing bacterial challenges by pathogens, such as *Haemophilus parasuis* and *Streptococcus suis*. Furthermore, pathologies of the liver appear interrelated with milk spots and bacteria-related pathologies, suggesting a potential multi-pathogen nature for these pathologies [23].

### 4.4. Regional Differences

The regional differences observed in the prevalence of the most frequent findings linked to the respiratory tract may reflect differences in the recording of pathological findings in the slaughterhouses [12,17]. However, to some extent, differences in the type (i.e., age and sex) and intensity (i.e., indoor intensive systems, outdoor free-range systems, etc.) of pig production and stocking density of agricultural holdings delivering to slaughterhouses in the specific region, in addition to seasonal effects, may also be relevant [12,30,45,46,47]. Among the major pig-producing federal states, pleuritis and pneumonia were more frequently reported in Styria, whereas foreign body in the lung was much more frequently registered in Upper and Lower Austria. This might reflect a lack of standardisation in the current meat inspection system [48] or may be a result of the two different types of software implemented for documenting pathological findings in meat inspection in Austria [49].

As only the location of the slaughterhouse and not that of the agricultural holding was available in this dataset, it cannot be excluded that frequent pathological findings in a slaughterhouse may also reflect the fact that animals have been transported for long distances or from farms with considerable herd health issues.

### 4.5. Potential Influencing Factors

Our analysis indicated that agricultural holding, slaughterhouse and quarter have a significant influence on the observed prevalence of meat inspection findings, which has also been reported in previous studies [12,19,50].

Overall, most variance in the prevalence can be explained by the agricultural holding (average R^2^ = 0.61), which may reflect differences in the type of pigs sent for slaughter, prevalence of specific diseases, farm management, type of housing, part or full-time occupation of farmers, size of holding and overall biosecurity level, which could be beneficial to herd health [11,45,46,47]. A clustering approach using meat inspection data from two Dutch slaughterhouses previously highlighted differences between groups of farms by lung abnormalities on the basis of data characteristics involving incidence, time pattern and degree of autocorrelation [48].

Furthermore, the slaughterhouse had a major impact on the variance of prevalence of pathological findings (R^2^ = 0.19). This may be explained by the heterogeneous performance of Austrian slaughterhouses, as previously described [17], but also by the current method of recording the findings as this may be prone to inconsistencies during the meat inspection process. A Danish study comparing routine meat inspection data with those collected at systematic health monitoring concluded that caution should be used whenever routine meat inspection data are used for purposes other than those for which they were originally intended. Results collected at one slaughterhouse highlighted moderate correlation for pleuritis and lung lesions, but poor correlation for pericarditis, which could only partly be explained by the type of meat inspection conducted at the abattoir [51].

Finally, the quarter also plays a role in terms of influencing the frequency of pathological findings (R^2^ = 0.05). This is in agreement with several reports highlighting the seasonality of respiratory tract infection [52].

Residuals demonstrate a lack of additional information and would decrease if further data were available, e.g., on the type of pigs, the farms of origin, the individual slaughter batch or the individual meat inspectors.

In Category 1, the agricultural holdings (R^2^_AH_ ranged from 0.08 to 0.66) had a significant impact on the recorded prevalence of pathological findings, but this is also true for the slaughterhouses. Interestingly, in two of the large federal states (Upper and Lower Austria), the influence of the factor slaughterhouse surpassed that of the factor agricultural holding (R^2^_SH_LA_ = 0.47, R^2^_SH_UA_ = 0.35). This is very relevant, as these slaughterhouses represent 57.9% (n = 2,665,304) of all pig carcases inspected in 2016. By contrast, in Styria, where most pigs were slaughtered, the agricultural holding (R^2^ = 0.33) explained more variability of findings in Category 1 than the slaughterhouses (R^2^ = 0.26), although the slaughterhouse contribution was still quite considerable. These findings confirm observations from previous studies highlighting both the value of slaughterhouse data for feedback systems to farmers [31] as well as the need for improved recording procedures in the slaughterhouses as these introduce considerable variability, which may hamper the use of meat inspection data for a reliable feedback system to farmers [48].

### 4.6. Variability within the Slaughterhouses

Differences between slaughterhouses could have been analysed in more detail by assessing the meat inspectors individually [17]. However, since the inter-rater disagreement was not assessable with the available data, homogeneity of the group variance was assessed. Our results indicate that the slaughterhouses differed significantly from each other. These differences in meat inspection amongst the various slaughterhouses may lead to a more or less favourable outcome for the farmer. As only the classification and the weight of the carcase without the organs are used to calculate the price paid to the farmer, the recorded lesions (D-codes) which may lead to extensive trimming only slightly impact the price a producer receives. Most meat inspection findings merely serve as an advisory feedback system for farm management, and are currently likely to be neglected or overlooked. Furthermore, if the documentation is not precise and comparable this can contribute greatly to an observed discrepancy in meat inspection outcomes.

In addition, these significant observations cannot exclusively be explained by the difference amongst the various slaughterhouses, but also due to the differences within the slaughterhouses (e.g., meat inspector). The analysis in four out of six federal states with several slaughterhouses, including the three states slaughtering most animals and having the largest number of slaughterhouses, indicated that the significant impact of slaughterhouses cannot only be attributed to the difference amongst each other but also to inconsistencies within the slaughterhouses, particularly with respect to Category 1 and 2 (see *p*- and H-value < 0.05 in Table 3). As these two categories make up the vast majority of findings in this study, this underlines the need for improved harmonisation of recording meat inspection data. Inconsistencies in recorded findings by the individual meat inspectors are likely since there is currently no further specification for each code defined in national legislation for documenting lesions. It must be assumed that this hampers comparable and reproducible recording in meat inspection. Unfortunately, further investigation of this aspect of meat inspection systems was beyond the scope of this study. Ghidini and coworkers highlighted in their studies that post-mortem inspection is prone to error and that to achieve a high level of standardisation and to obtain consistent data, it is necessary to develop standard operating procedures and to thoroughly train all inspectors involved for obtaining consistent data [39].

### 4.7. Limitations

There are several limitations concerning the dataset used here and the approach taken to analyse these data. With respect to misclassification, the pathological finding “Foreign body in the lung” (E191) was grouped in Category 1 in this analysis, as it is not necessarily clearly associated with abnormalities due to slaughter technique, and it was assumed that the code reflects pathological occurrences originating pre-slaughter. However, a major problem in meat inspection is scalding water in the lung which can be confused with a lesions due to pneumonia as they are not always easily distinguishable on the surface [15]. As slaughterhouses in Austria are of smaller size and lungs do not play an important role in meat marketing, horizontal scalding is still the most frequent method in Austria. Correlation analysis did not show any association with either of the other pathologies of the respiratory system nor slaughter-technique-associated abnormalities, which supports the assumption that better understanding of the underlying condition is needed (Supplementary Figure S2). To distinguish clearly between these two features, a separate meat inspection code for scalding water in the lung should be established and precise distinction ensured by cutting the distal third of either of the large lung lobes. Correlation between the two D-codes pertaining to foreign body in the lung (namely, D140-Foreign body & D190-Abnormalities due to certain slaughter techniques) might also reflect that both codes should be allocated to Category 5, and not one to Category 4 as there was no correlation with any other animal-health-related factor.

With respect to the dataset, it was beneficial that the number of animals investigated, the number of findings per code and the number of animals with findings were available for each agricultural holding. As several findings may have been documented for an individual animal, summary figures for findings per category might have overestimated the real situation, but this was considered negligible for the overall picture. However, it would have been preferable to have obtained further data for individual slaughter batches rather than just for quarterly deliveries, which limited consideration of individual problems or seasonal patterns at the farm level.

Unfortunately, for reasons of data protection, no further information was available for this analysis on the agricultural holding of origin, and data were also lacking as to whether all animals within a consignment originated from the same farm, as the recorded unit may also reflect a livestock trader or short-term stay in a holding. This could complicate interpretation of the farm-related influence on the variability of results. For example, no information was available on the age of the animals in this dataset, although such data were available at the slaughterhouse level. It has been described that additional information such as production system or age at slaughter (e.g., reflected by weight) could also increase R^2^ within the model as this has significant influence in the occurrence of respiratory-tract-related findings [19]. Furthermore, this lack of detailed information hampers the drafting of useful feedback for the individual delivery and farm of origin. Currently, farmers receive a list of recorded findings for each delivery, but no interpretation of the results or summary indicators.

Other pathologies are not reflected in the current meat inspection in Austria. For example, it has been stated that stomach complaints, such as ulcer-associated findings, are also of great significance in pig health with a prevalence of approximately 80% [53].

It is noteworthy that two factors in our model, namely, agricultural holding and slaughterhouse, may have considerable interactions with each other, i.e., one agricultural holding delivered pigs to different slaughterhouses. Therefore, in the federal states Lower Austria, Upper Austria and Styria, we additionally included a separate model where the interactions of these factors were included as cross-classified random factors. Nevertheless, the corresponding results did not differ considerably from other model results presented here.

## 5. Conclusions

Although Regulation (EU) 2014/218 has laid down the possibility of only performing visual meat inspection in pigs, many countries within the EU still run a traditional meat inspection. This is mainly to successfully compete in international trade, as many countries outside the EU still require certain traditional methods [54]. This may be controversial since the advantages in marketing meat for the international market, due to the application of methods not required by European law, is still requested by stake holders (e.g., third countries) and provided by public administration (e.g., incision of lymph nodes).

Our analysis of such a high number of slaughter records covering all slaughterhouses in the country supports improvement of the recording system in Austria, as well as allowing future analysis of the progress made. Further harmonisation of recordings is challenging but urgently needed. Our analysis shows that data contain relevant information on the impact of agricultural holdings on the prevalence of pathological findings and that the current form of documentation leaves room for improvement. Correct documentation of pathological findings should ensure valid prevalence estimates collected at each slaughterhouse and adequate payment of farmers for the pigs delivered, regardless of where the animals have been slaughtered. Nevertheless, it must be taken into account that the current dataset originates from an early stage of the digital documentation in meat inspection in Austria.

To meet the demand for a valid feedback system to farmers and attending veterinarians, a robust and more detailed recording of frequent pathological findings, especially those regarding the respiratory tract and the liver, should be developed in the future. Through such a feedback system, the assessment of herd health could be more accurate and thereby farm managers and herd veterinarians could benefit greatly in their efforts to improve herd health and pork quality [55]. Additionally, continuous incorporation of feedback from slaughterhouses into herd management is more likely to improve farm performance over time [9] and to support the evaluation of the efficiency of measures taken. Our results confirm the need for an improved and standardised meat inspection recording system, which delivers precise, comparable and reproducible results, as currently the livestock holders simply receive a summation of the codes documented with no further explanation. The feedback must reflect upon the current health status from the consignment slaughtered in order to be beneficial for the stakeholders in meat production. However, the exact adjustments and to what extent alterations in the existing meat inspection system have to be made, has yet to be evaluated and will be the topic of further investigations.

## Figures and Tables

**Table 1:** Absolute number (n = 3,609,619) and prevalence (%) of the main pathological findings (more than 0.01% of all findings) stratified by the five categories.

Pathological Finding (Code) Stratified by Five Main Categories #	Absolute Number of Pathological Findings	Prevalence (Per Total Slaughtered Animals) ^1^ (%)	Prevalence (Per Total Pathological Findings) ^2^ (%)
Pleuritis (D169)	58,619	1.27	1.62
Pneumonia (E167)	1,009,236	21.91	27.96
Pericarditis (E168)	107,400	2.33	2.98
Pleuritis (E169)	319,754	6.94	8.86
Pluck adhesion (E169gs)	61,598	1.34	1.71
Foreign body in the lung (E191)	669,196	14.53	18.54
**Total: Category 1**	**2,225,803**	**48.33**	**61.66**
Peritonitis (D169a)	2747	0.06	0.08
Milk spots (E085)	918,112	19.94	25.44
Pathologies regarding the kidney (E163ni)	18,081	0.39	0.5
Fatty liver (E165)	4594	0.1	0.13
Hepatitis, Perihepatitis (E166)	151,337	3.29	4.19
Peritonitis (E169a)	1509	0.03	0.04
**Total: Category 2**	**1,096,698**	**23.81**	**30.38**
Polyarthritis (D061)	16,326	0.35	0.45
Multiple abscesses (D164)	23,784	0.52	0.66
Cachexia (D170)	1002	0.02	0.03
Singular arthritis (E061)	26,115	0.57	0.72
Skin parasites (E087)	11,773	0.26	0.33
Isolated singular abscess (E164)	45,299	0.98	1.25
**Total: Category 3**	**124,489**	**2.7**	**3.45**
**Total: Category 4**	**57,838**	**1.26**	**1.6**
**Total: Category 5**	**104,791**	**2.28**	**2.9**

# Categories: (1) pathologies regarding the respiratory system and the heart, (2) pathologies regarding the abdominal organs, (3) pathologies regarding the skin and the locomotor system, (4) other pathologies and (5) slaughter-technique-induced abnormalities ^1^ Absolute number of pathological findings compared to total number of slaughtered animals (n = 4,604,716). ^2^ Absolute number of pathological findings compared to total number of pathological findings (n = 3,609,619). Details for Category 3–5, which were all <1%, can be found in Supplementary Table S1. The totals by category are highlighted in bold to ease reading the table.

**Table 2:** Geographical distribution of prevalence (in %) of the pathological findings related to the respiratory system and heart (category 1) per federal state.

Federal State	Animals Tested	Pleuritis	Pneumonia	Pericarditis	Pleuritis	Pluck Adhesion	Foreign Body in the Lung
		(D169)	(E167)	(E168)	(E169)	(E169gs)	(E191)
		(in %)	(in %)	(in %)	(in %)	(in %)	(in %)
**B**	24,075	1.4	17.3	1.8	1.4	0.0	50.3
**C**	128,216	0.0	39.0	3.8	2.8	1.5	37.6
**LA**	982,712	0.1	14.6	2.0	6.5	1.1	24.5
**UA**	1,682,238	0.2	18.7	2.0	6.3	1.8	12.3
**SB**	29,663	0.0	7.8	1.2	1.5	1.5	29.0
**ST**	1,740,230	3.0	28.2	2.8	8.3	1.1	8.7
**T**	11,358	4.1	29.8	2.9	5.9	1.4	24.5
**V**	6224	0.1	5.3	3.6	2.1	0.1	0.2
**Total**	**4,604,716**	**1.3**	**21.9**	**2.3**	**6.9**	**1.3**	**14.5**

B: Burgenland; C: Carinthia; LA: Lower Austria; UA: Upper Austria; SB: Salzburg; ST: Styria; T: Tyrol; V: Vorarlberg. The headings and the total is highlighted in bold to make reading the table easier.

**Table 3:** PERMANOVA results and multivariate homogeneity of group dispersions (variances) of the prevalence of pathological findings with respect to the potential influence of slaughterhouse (SH), quarter (Q), agricultural holdings (AH) and residuals (Res.) stratified by the 5 main categories and federal state. Significant results are highlighted in green.

		Category 1			Category 2			Category 3			Category 4			Category 5		
		R^2^	*p*	H	R^2^	*p*	H	R^2^	*p*	H	R^2^	*p*	H	R^2^	*p*	H
**V**	**SH**	0.09	**<0.05**	0.90	0.02	**<0.05**	**<0.05**	na	na	na	na	na	na	na	na	na
	**Q**	0.05	**<0.05**	0.16	0.00	0.67	0.07	0.11	0.06	0.87	0.18	**<0.05**	0.59	0.05	0.60	0.17
	**AH**	0.66	**<0.05**	0.81	0.71	**<0.05**	0.88	0.72	0.20	0.82	0.81	**<0.05**	**<0.05**	0.78	0.29	0.47
	**Res.**	0.19			0.26			0.17			0.01			0.18		
**T**	**SH**	na	na	na	na	na	na	na	na	na	na	na	na	na	na	na
	**Q**	0.10	**<0.05**	0.47	0.19	**<0.05**	**<0.05**	0.09	**<0.05**	0.95	0.19	0.07	0.31	0.21	**<0.05**	**<0.05**
	**AH**	0.60	**<0.05**	0.15	0.47	0.78	0.13	0.71	**<0.05**	0.16	0.75	0.33	**<0.05**	0.58	**<0.05**	**<0.05**
	**Res.**	0.30			0.34			0.20			0.06			0.22		
**ST**	**SH**	0.26	**<0.05**	**<0.05**	0.15	**<0.05**	**<0.05**	0.24	**<0.05**	**<0.05**	0.38	**<0.05**	**<0.05**	0.18	**<0.05**	**<0.05**
	**Q**	0.00	**<0.05**	**<0.05**	0.00	**<0.05**	0.48	0.00	**<0.05**	**<0.05**	0.01	**<0.05**	**<0.05**	0.00	**<0.05**	**<0.05**
	**AH**	0.33	**<0.05**	**<0.05**	0.47	**<0.05**	**<0.05**	0.30	**<0.05**	**<0.05**	0.26	**<0.05**	**<0.05**	0.31	**<0.05**	**<0.05**
	**Res.**	0.41	-		0.38			0.45			0.36			0.51		
**LA**	**SH**	0.47	**<0.05**	**<0.05**	0.15	**<0.05**	**<0.05**	0.12	**<0.05**	**<0.05**	0.19	**<0.05**	**<0.05**	0.20	**<0.05**	**<0.05**
	**Q**	0.00	**<0.05**	0.87	0.00	0,19	**<0.05**	0.00	**<0.05**	0.21	0.00	**<0.05**	0.79	0.00	**<0.05**	**<0.05**
	**AH**	0.23	**<0.05**	na	0.47	**<0.05**	na	0.45	**<0.05**	**<0.05**	0.55	**<0.05**	**<0.05**	0.43	**<0.05**	**<0.05**
	**Res.**	0.30			0.38			0.43			0.25			0.37		
**UA**	**SH**	0.35	**<0.05**	**<0.05**	0.15	**<0.05**	**<0.05**	0.11	**<0.05**	**<0.05**	0.32	**<0.05**	**<0.05**	0.24	**<0.05**	**<0.05**
	**Q**	0.00	**<0.05**	**<0.05**	0.00	**<0.05**	0.55	0.00	**<0.05**	**<0.05**	0.00	**<0.05**	**<0.05**	0.00	**<0.05**	**<0.05**
	**AH**	0.25	**<0.05**	**<0.05**	0.41	**<0.05**	na	0.45	**<0.05**	**<0.05**	0.43	**<0.05**	**<0.05**	0.44	**<0.05**	**<0.05**
	**Res.**	0.40			0.44			0.44			0.25			0.31		
**C**	**SH**	0.28	**<0.05**	**<0.05**	0.48	**<0.05**	0.056	0.03	**<0.05**	**<0.05**	0.11	**<0.05**	0.29	0.06	**<0.05**	0.85
	**Q**	0.01	**<0.05**	0.68	0.01	**<0.05**	0.086	0.14	**<0.05**	0.60	0.03	**<0.05**	0.23	0.04	**<0.05**	0.09
	**AH**	0.42	**<0.05**	**<0.05**	0.27	**<0.05**	**<0.05**	0.50	**<0.05**	**<0.05**	0.62	**<0.05**	**<0.05**	0.69	0.31	0.29
	**Res.**	0.30			0.24			0.33	-		0.24			0.21		
**S**	**SH**	na	na	na	na	na	na	na	na	na	na	na	na	na	na	na
	**Q**	0.03	**<0.05**	**<0.05**	0.00	0.23	0.23	0.00	0.65	0.73	0.01	0.13	0.73	0.05	0.07	0.63
	**AH**	0.55	**<0.05**	**<0.05**	0.77	**<0.05**	0.97	0.79	**<0.05**	0.22	0.83	**<0.05**	0.86	0.90	0.30	0.19
	**Res.**	0.42			0.23			0.21			0.17			0.06		
**B**	**SH**	0.08	**<0.05**	0.09	na	na	na	0.16	**<0.05**	**<0.05**	0.16	**<0.05**	0.33	0.15	**<0.05**	0.52
	**Q**	0.00	0.22	0.65	0.00	0.68	0.23	0.02	0.01	0.61	0.04	**<0.05**	**<0.05**	0.06	0.13	0.57
	**AH**	0.63	**<0.05**	0.15	0.70	**<0.05**	0.69	0.67	0.05	0.17	0.73	**<0.05**	0.22	0.76	0.11	**<0.05**
	**Res.**	0.29			0.30			0.16			0.08			0.02		

R^2^ = R squares (explained variance); *p* = *p*-value, significance level of PERMANOVA; H = significance level (*p*-value) of homogeneity of the group variances; a value ≥ 0.05 indicates homogeneity of group disperations; na = not applicable because only one or two slaughterhouses or no pathology records in a specific category were available. Federal states: V: Vorarlberg; T: Tyrol; ST: Styria; LA Lower Austria; UA: Upper Austria; C: Carinthia; SB: Salzburg; B: Burgenland. Important findings are highlighted in bold to make reading the table easier.

## Data Availability

Restrictions apply to the availability of these data. Data was obtained from Federal Ministry of Social Affairs, Health, Care and Consumer Protection and are available from the authors with the permission of Federal Ministry of Social Affairs, Health, Care and Consumer Protection only.

## Supplementary Materials

The following are available online at https://www.mdpi.com/article/10.3390/ani11051442/s1, Figure S1: Distribution of the number of agricultural holdings per slaughterhouse; Figure S2: Spearman Correlation between the prevalence of individual pathological findings; Table S1: Absolute number (n = 3,609,619) and prevalence (%) of main pathological findings (more than 0.01% of all findings) stratified by the five main categories #.

## References

[1] Rieper S.M. (2013). Epidemiologische Untersuchungen zur Verwendung der tierärztlichen Befundung am Schlachthof als tierorientierte Tierschutzkriterien zur Beurteilung der Tiergesundheit und des Tierwohls der Tiere in Schweinemastbeständen. Dissertation Thesis.

[2] Schuh M., Köfer J., Fuchs K. (2000). Errichtung eines Rückmeldesystems zur Kontrolle der Tiergesundheit–Häufigkeit von Organbefunden und deren ökonomische Relevanz bei Schlachtschweinen. Wien. Tierarztl. Monatsschr..

[3] Stärk K.D.C., Alonso S., Dadios N., Dupuy C., Ellerbroek L., Georgiev M., Hardstaff J., Huneau-Salaün A., Laugier C., Mateus A. (2014). Strengths and weaknesses of meat inspection as a contribution to animal health and welfare surveillance. Food Control.

[4] Boessen C.R., Kliebenstein J.B., Cowart R.P., Moore K.C., Burbee C.R. (1988). Effective use of slaughter checks to determine economic losses from morbidity in swine. Acta Vet. Scand. Suppl..

[5] Wellenberg G.J., Bouwkamp F.T., Wolf P.J.v.d., Swart W.A.J.M., Mombarg M.J., de Gee A.L.W. (2010). A study on the severity and relevance of porcine circovirus type 2 infections in Dutch fattening pigs with respiratory diseases. Vet. Microbiol..

[6] Teixeira D.L., Harley S., Hanlon A., O’Connell N.E., More S.J., Manzanilla E.G., Boyle L.A. (2016). Study on the association between tail lesion score, cold carcass weight, and viscera condemnations in slaughter pigs. Front. Vet. Sci..

[7] Straw B.E., Shin S.J., Yeager A.E. (1990). Effect of pneumonia on growth rate and feed efficiency of minimal disease pigs exposed to *Actinobacillus pleuropneumoniae* and *Mycoplasma hyopneumoniae*. Prev. Vet. Med..

[8] Ferraz M.E.S., Almeida H.M.S., Storino G.Y., Sonálio K., Souza M.R., Moura C.A.A., Costa W.M.T., Lunardi L., Linhares D.C.L., de Oliveira L.G. (2020). Lung consolidation caused by *Mycoplasma hyopneumoniae* has a negative effect on productive performance and economic revenue in finishing pigs. Prev. Vet. Med..

[9] Sanchez-Vazquez M.J., Smith R.P., Kang S., Lewis F., Nielen M., Gunn G.J., Edwards S.A. (2010). Identification of factors influencing the occurrence of milk spot livers in slaughtered pigs: A novel approach to understanding *Ascaris suum* epidemiology in British farmed pigs. Vet. Parasitol..

[10] Holt H.R., Alarcon P., Velasova M., Pfeiffer D.U., Wieland B. (2011). BPEX Pig Health Scheme: A useful monitoring system for respiratory disease control in pig farms?. BMC Vet. Res..

[11] Laanen M., Persoons D., Ribbens S., de Jong E., Callens B., Strubbe M., Maes D., Dewulf J. (2013). Relationship between biosecurity and production/antimicrobial treatment characteristics in pig herds. Vet. J..

[12] Elbers A.R.W., Tielen M.J.M., Snijders J.M.A., Cromwijk W.A.J., Hunneman W.A. (1992). Epidemiological studies on lesions in finishing pigs in the Netherlands. I. Prevalence, seasonality and interrelationship. Prev. Vet. Med..

[13] Hoischen-Taubner S., Blaha T., Werner C., Sundrum A. (2011). Repeatability of anatomical-pathological findings at the abattoir for characteristics of animal health. Arch. Lebensmittelhyg..

[14] Eckhardt P., Fuchs K., Kornberger B., Kofer J. (2010). Slaughter findings feedback systems—Its use for farms of origin? Schlachtbefundruckmeldesysteme—Nutzen für die primärproduktion?. Berl. Munch. Tierarztl. Wochenschr..

[15] Nathues H., Hewicker-Trautwein M., Beilage E.G. (2008). Differenzierung Schlachtungsbedingter Artefakte von Pneumonischen Veränderungen beim Lungencheck an Schlachtschweinen. Tierarztl. Prax. Ausgabe G Grosstiere-Nutztiere.

[16] Bonde M., Toft N., Thomsen P.T., Sørensen J.T. (2010). Evaluation of sensitivity and specificity of routine meat inspection of Danish slaughter pigs using Latent Class Analysis. Prev. Vet. Med..

[17] Schleicher C., Scheriau S., Kopacka I., Wanda S., Hofrichter J., Köfer J. (2013). Analysis of the variation in meat inspection of pigs using variance partitioning. Prev. Vet. Med..

[18] Correia-Gomes C., Eze J.I., Borobia-Belsué J., Tucker A.W., Sparrow D., Strachan D., Gunn G.J. (2017). Voluntary monitoring systems for pig health and welfare in the UK: Comparative analysis of prevalence and temporal patterns of selected non-respiratory post mortem conditions. Prev. Vet. Med..

[19] Scollo A., Gottardo F., Contiero B., Mazzoni C., Leneveu P., Edwards S.A. (2017). Benchmarking of pluck lesions at slaughter as a health monitoring tool for pigs slaughtered at 170 kg (heavy pigs). Prev. Vet. Med..

[20] Kongsted H., Sørensen J.T. (2017). Lesions found at routine meat inspection on finishing pigs are associated with production system. Vet. J..

[21] Merialdi G., Dottori M., Bonilauri P., Luppi A., Gozio S., Pozzi P., Spaggiari B., Martelli P. (2012). Survey of pleuritis and pulmonary lesions in pigs at abattoir with a focus on the extent of the condition and herd risk factors. Vet. J..

[22] Steinmann T., Blaha T., Meemken D. (2014). A simplified evaluation system of surface-related lung lesions of pigs for official meat inspection under industrial slaughter conditions in Germany. BMC Vet. Res..

[23] Sanchez-Vazquez M.J., Nielen M., Gunn G.J., Lewis F.I. (2012). National monitoring of *Ascaris suum* related liver pathologies in English abattoirs: A time-series analysis, 2005–2010. Vet. Parasitol..

[24] Auer H. (2005). Die Trichinellose des menschen in Österreich. Wien. Tierarztl. Monatsschr..

[25] Food Safety Authority E., Boelaert F., Stoicescu A., Amore G., Messens W., Hempen M., Rizzi V., Antoniou S.-E., Baldinelli F., Dorbek-Kolin E. (2019). The European Union One Health 2019 Zoonoses Report. EFSA J..

[26] QS-Report. https://www.q-s.de/flip/QS-Report-Fleisch-Fleischwaren-2-2016/files/assets/basic-html/page-1.html.

[27] Anderson J.M. (2017). Permutational Multivariate Analysis of Variance (PERMANOVA). Wiley StatsRef: Statistics Reference Online. https://onlinelibrary.wiley.com/doi/full/10.1002/9781118445112.stat07841.

[28] Martinez Arbizu P. pairwiseAdonis: Pairwise Multilevel Comparison Using Adonis. https://github.com/pmartinezarbizu/pairwiseAdonis#citation.

[29] Oksanen J., Blanchet F.G., Kindt R., Legendre P., Minchin P.R., O’Hara R.B., Simpson G.L., Solymos P., Stevens M.H.H., Wagner H. (2014). Vegan: Community Ecology Package. R Package Version 2.2-0. https://cran.r-project.org/web/packages/vegan/index.html.

[30] Statistic Austria. http://www.statistik.at/web_de/statistiken/menschen_und_gesellschaft/gesundheit/krebserkrankungen/dickdarm_enddarm/index.html.

[31] Guardone L., Vitali A., Fratini F., Pardini S., Goga B.T.C., Nucera D., Armani A. (2020). A retrospective study after 10 years (2010–2019) of meat inspection activity in a domestic swine abattoir in tuscany: The slaughterhouse as an epidemiological observatory. Animals.

[32] Gottschalk M. (2015). The challenge of detecting herds sub-clinically infected with *Actinobacillus pleuropneumoniae*. Vet. J..

[33] Garcia-Diez J., Coelho A.C. (2014). Causes and factors related to pig carcass condemnation. Vet. Med..

[34] NADIS Animal Health Skills. https://www.nadis.org.uk/disease-az/pigs/whole-carcass-condemnation/.

[35] Ceccarelli M., Leprini E., Sechi P., Iulietto M.F., Grispoldi L., Goretti E., Cenci-Goga B.T. (2018). Analysis of the causes of the seizure and destruction of carcasses and organs in a slaughterhouse in central Italy in the 2010–2016 period. Ital. J. Food Saf..

[36] Grosse Beilage E., Wendt M., Wendt M. (2013). Diagnostik und Gesundheitsmanagement im Schweinebestand.

[37] Pill K., Blaha T., Richter T. (2013). Erfassung und Analyse tierbezogener klinischer und pathologischer/anatomischer Befunde bei Schweinen am Schlachthof. Rundschau für Fleischhygiene und Leb..

[38] Alban L., Petersen J.V., Busch M.E. (2015). A comparison between lesions found during meat inspection of finishing pigs raised under organic/free-range conditions and conventional, indoor conditions. Porc. Health Manag..

[39] Ghidini S., Zanardi E., Di Ciccio P.A., Borrello S., Belluzi G., Guizzardi S., Ianieri A. (2018). Development and test of a visual-only meat inspection system for heavy pigs in Northern Italy. BMC Vet. Res..

[40] Griglio B., Sattanino G., Siviero T., Guarda F. (2004). Valutazioni Sulle Patologie Rilevate Nei Suini Macellati in Piemonte. Large Anim. Rev..

[41] Stewart T.B., Hoyt P.G., Straw B.E., Zimmerman J.J., D’Allaire S., Taylor D.J. (2006). Internal parasites. Diseases of Swine.

[42] Kreinoecker K., Sattler T., Hagmueller W., Hennig-Pauka I., Schmoll F. (2017). NoOccurrence of antibodies against toxoplasma, leptospira and prrsv and the incidence of salmonella and ascaris suum on organic pig fattening farms in Austria. Wiener Tierarztl. Monatsschrift.

[43] Bernardo T.M., Dohoo I.R., Ogilvie T. (1990). A critical assessment of abattoir surveillance as a screening test for swine ascariasis. Can. J. Vet. Res..

[44] Alban L., Steenberg B., Stephensen F.T., Olsen A., Petersen J.V. (2011). Overview on current practices of meat inspection in the EU. EFSA Support. Publ..

[45] Heinonen M., Gróhn Y.T., Saloniemi H., Eskola E., Tuovinen V.K. (2001). The effects of health classification and housing and management of feeder pigs on performance and meat inspection findings of all-in-all-out swine-finishing herds. Prev. Vet. Med..

[46] Meyns T., Van Steelant J., Rolly E., Dewulf J., Haesebrouck F., Maes D. (2011). A cross-sectional study of risk factors associated with pulmonary lesions in pigs at slaughter. Vet. J..

[47] Fraile L., Alegre A., López-Jiménez R., Nofrarías M., Segalés J. (2010). Risk factors associated with pleuritis and cranio-ventral pulmonary consolidation in slaughter-aged pigs. Vet. J..

[48] Hulsegge B., de Greef K.H., Hulsegge I. (2018). A time-series approach for clustering farms based on slaughterhouse health aberration data. Prev. Vet. Med..

[49] Trockenbacher A. (2017). Elektronische Befunderfassung im Rahmen der Schlachttier- und Fleischuntersuchung in österreichischen Schlachtbetrieben. Diploma Thesis.

[50] Enøe C., Christensen G., Andersen S., Willeberg P. (2003). The need for built-in validation of surveillance data so that changes in diagnostic performance of post-mortem meat inspection can be detected. Prev. Vet. Med..

[51] Nielsen S.S., Nielsen G.B., Denwood M.J., Haugegaard J., Houe H. (2015). Comparison of recording of pericarditis and lung disorders at routine meat inspection with findings at systematic health monitoring in Danish finisher pigs. Acta Vet. Scand..

[52] Cleveland-Nielsen A., Nielsen E.O., Ersboll A.K. (2002). Chronic pleuritis in Danish slaughter pig herds. Prev. Vet. Med..

[53] Swaby H., Gregory N.G. (2012). A note on the frequency of gastric ulcers detected during post-mortem examination at a pig abattoir. Meat Sci..

[54] Hansen R.K., Nielsen L.H., El Tholth M., Haesler B., Foddai A., Alban L. (2018). Comparison of Alternative Meat Inspection Regimes for Pigs From Non-Controlled Housing–Considering the Cost of Error. Front. Vet. Sci..

[55] Permentier L., Maenhout D., Deley W., Broekman K., Vermeulen L., Agten S., Verbeke G., Aviron J., Geers R. (2015). Lung lesions increase the risk of reduced meat quality of slaughter pigs. Meat Sci..

