# Supplementary material for: Agricultural Holdings and Slaughterhouses’ Impact on Patterns of Pathological Findings Observed during Post-Mortem Meat Inspection
Source: Animals (Basel). 2021 May 18;11(5):1442. doi: 10.3390/ani11051442 (PMC8157594; doi:10.3390/ani11051442)

Supplementary Figure S2.: Spearman Correlation between the prevalence of individual pathological findings

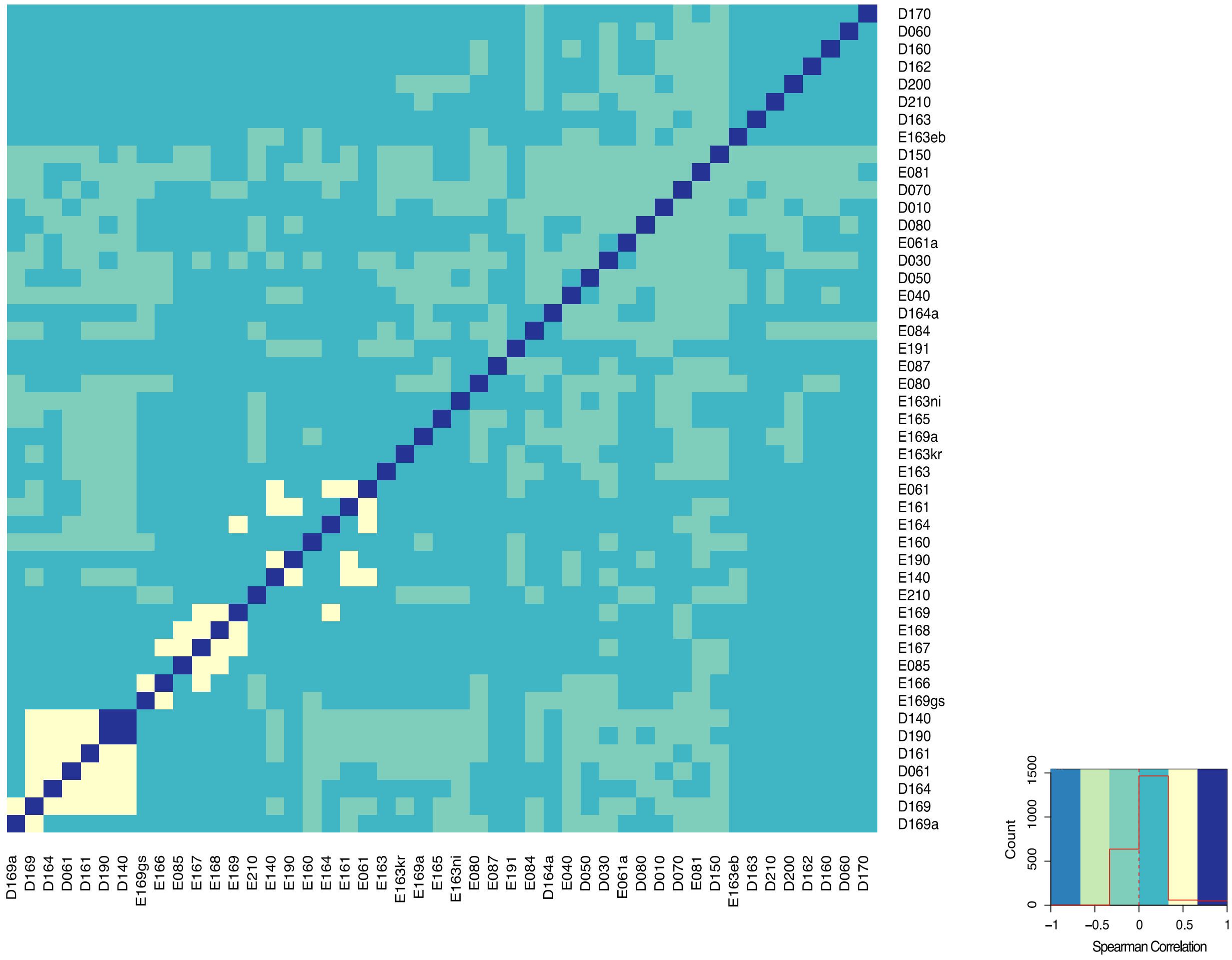

Supplement: Supplementary file 1 [file animals-11-01442-s001.zip › PAPER_Figure S2.pdf]
