# Supplementary material for: Agricultural Holdings and Slaughterhouses’ Impact on Patterns of Pathological Findings Observed during Post-Mortem Meat Inspection
Source: Animals (Basel). 2021 May 18;11(5):1442. doi: 10.3390/ani11051442 (PMC8157594; doi:10.3390/ani11051442)
Supplement: Supplementary file 1 [file animals-11-01442-s001.zip › PAPER_Table S1_18042021.pdf]

Supplementary Table S1: Absolute number (n=3,609,619) and prevalence [in %] of main pathological findings (more than 0.01% of all findings) stratified by the five main categories[#]:

Pathological finding (Code) stratified by five main categories	Absolute number of pathological findings	Prevalence (per total slaughtered animals)[1] [%]	Prevalence (per total pathological findings)[2] [%]
Pleurisy (D169)	58,619	1.27	1.62
Pneumonia (E167)	1,009,236	21.91	27.96
Pericarditis (E168)	107,400	2.33	2.98
Pleurisy (E169)	319,754	6.94	8.86
Pluck adhesion (E169gs)	61,598	1.34	1.71
Foreign body in the lung (E191)	669,196	14.53	18.54
**Total: Category 1**	**2,225,803**	**48.33**	**61.66**
Peritonitis (D169a)	2,747	0.06	0.08
Milk spots (E085)	918,112	19.94	25.44
Pathologies regarding the kidney (E163ni)	18,081	0.39	0.5
Fatty liver (E165)	4,594	0.1	0.13
Hepatitis, Perihepatitis (E166)	151,337	3.29	4.19
Peritonitis (E169a)	1,509	0.03	0.04
**Total: Category 2**	**1,096,698**	**23.81**	**30.38**
Polyarthritis (D061)	16,326	0.35	0.45
Multiple abscesses (D164)	23,784	0.52	0.66
Cachexia (D170)	1,002	0.02	0.03
Singular arthritis (E061)	26,115	0.57	0.72
Skin parasites (E087)	11,773	0.26	0.33
Isolated singular abscess (E164)	45,299	0.98	1.25
**Total: Category 3**	**124,489**	**2.7**	**3.45**
Foreign body (D140)	16,361	0.36	0.45
Odor deviation (D163)	2,165	0.05	0.06
Isolated pathophysiological changes, consistency and or organoleptic abnormalities (E160)	16,041	0.35	0.44
Odor deviation (E163)	912	0.02	0.03
Boar (E163eb)	4,235	0.09	0.12
Cryptorchidic (E163kr)	3,451	0.07	0.1
Parts that pose a potential risk to human health according to the official veterinarian (E210)	13,371	0.29	0.37
**Total: Category 4**	**57,838**	**1.26**	**1.6**
Parts that are soaked in blood or water (D161)	14182	0.31	0.39
Abnormalities due to certain slaughter techniques (Charring, machine damage, soiling etc.) (D190)	24,799	0.54	0.69
Contaminated meat parts or organs (E140)	21,453	0.47	0.59
Single parts that are soaked in blood or water (E161)	18,235	0.4	0.51
Single abnormalities due to certain slaughter techniques (E190)	26,079	0.57	0.72
**Total: Category 5**	**104,791**	**2.28**	**2.9**

[#] Categories: (1) pathologies regarding the respiratory system and the heart, (2) pathologies regarding the abdominal organs, (3) pathologies regarding the skin and the locomotor system, (4) other pathologies and (5) slaughter technique induced abnormalities

[1] Absolute number of pathological findings compared to total number of slaughtered animals (n= 4.604.716).

[2] Absolute number of pathological findings compared to total number of pathological findings (n= 3.609.619).
